# Evaluation of factors related to overdose in patients with impaired consciousness who are transported by emergency medical services: an age-specific research

**DOI:** 10.1186/s40780-021-00217-8

**Published:** 2021-10-01

**Authors:** Kazuki Nagashima, Megumi Sumida, Shoichi Imanaka, Tatsuro Kuwabara, Ichiro Kaneko, Yasufumi Miyake, Nobuhiro Yasuno, Fumio Itagaki, Machiko Watanabe

**Affiliations:** 1 Laboratory of Clinical Pharmaceutics, Faculty of Pharma-Science, Teikyo University, 2-11-1 Kaga, Itabashi-ku, Tokyo, 173-8605 Japan; 2 Department of Pharmacy, Teikyo University Hospital, 2-11-1 Kaga, Itabashi-ku, Tokyo, 173-8606 Japan; 3 Department of Emergency Medicine, School of Medicine, Teikyo University, 2-11-1 Kaga, Itabashi-ku, Tokyo, 173-8605 Japan

**Keywords:** Drug overdose, Psychotropic drug, Benzodiazepine, Antidepressant, Age, Female, Disorder of consciousness

## Abstract

**Background:**

Drug overdose accounts for most of the admissions to the emergency department. Prescription drugs, most of which are psychotropic medications, are often misused for drug overdose. The purpose of this study was to investigate the association between overdose in patients transported with disorders of consciousness and psychotropic medications administered prior to transport, so as to enable quick differentiation of drug overdose patients from patients with disorders of consciousness.

**Methods:**

We evaluated 222 patients transported to the Advanced Critical Care Center of Teikyo University Hospital due to disorders of consciousness. The patients were categorized into two groups: overdose group (*n* = 128) and control group with other disorders of consciousness (*n* = 94). Logistic regression models were used to assess the association between disorders of consciousness due to drug overdose and psychotropic drugs prescribed before emergency transportation based on sex and age.

**Results:**

According to multivariate logistic regression analysis, only female sex (odds ratio [OR] 4.54, 95% confidence interval [CI] 2.43–8.05, *P* < 0.0001) was associated with overall overdose. Results from the univariate logistic regression analysis showed that in the group of patients aged 40–50 years, female sex (OR 4.36, 95% CI; 1.54–12.4, *P* = 0.006) and the use of psychotropic drugs (OR 5.05, 95% CI; 1.75–14.6, *P* = 0.003), benzodiazepines (OR 4.64, 95% CI; 1.61–13.4, *P* < 0.05), antidepressants (OR 11.4, 95% CI; 2.35–55.8, *P* = 0.003), and anticonvulsants (OR 4.46, 95% CI; 1.11–17.9, *P* = 0.035) were associated with overdose. According to multivariate logistic regression analysis, female sex (OR 4.44, 95% CI; 1.37–14.3, *P* = 0.013) and antidepressants (OR 7.95, 95% CI; 1.21–52.1, *P* = 0.031) were associated with overdose patients aged 40–50 years.

**Conclusions:**

As a reference in distinguishing overdose in women in their 40s and 50s who present with impaired consciousness, attention may need to be paid to the type of psychotropic drug used, especially antidepressants.

## Background

Drug overdose is the excessive use of drugs, resulting in acute and deleterious effects on the body or psyche, and is one of the major causes of emergency transportation of patients [1–3]. The drugs prescribed by physicians, especially psychotropic drugs, such as benzodiazepine receptor agonists (BZs) and antidepressants [4–6], are misused by these patients for drug overdose. In addition, more than 75% of these cases include those who intentionally overdose for suicidal purposes rather than accidental overdose [6, 7].

BZs have been reported to be the most common psychotropic drugs with a tendency for overdose [8] and are associated with an increased risk of suicide [9–11]. Furthermore, it has been reported that as with abuse of illegal drugs, such as stimulants, the use of BZs is associated with certain patient characteristics, including younger age and female sex [12]. In addition, significant increases in the prevalence of BZ exposure and overdose have been observed, especially in children [13]. In other reports, acute BZ intoxication and accidental overdose have been reported in elderly people [14–16]. Furthermore, some studies have reported that antidepressants are more commonly used for intentional drug overdose in women than in men [17–19]. Depression is a common risk factor associated with more than half of suicidal deaths [20]. Therefore, treatment of depression is often a target of suicide prevention efforts. Furthermore, there are reports of iatrogenic suicidal effects of antidepressants [21, 22] as well as the potential lethality of antidepressant overdose [23].

Disorders of consciousness are frequently encountered symptoms in the emergency department, and sudden disorders of consciousness are often caused by brain or heart angiopathy, arrhythmias, and seizures, or from life-threatening infections of the lungs and liver as well as drug-induced dysfunction of organs, such as the kidneys. For differential diagnosis of overdose, there is a need to exclude other diseases that disturb consciousness (in order). Therefore, it may take some time to confirm a diagnosis as an overdose. There is therefore the need for prompt identification of overdose patients and subsequent identification of the responsible drugs. Identifying the responsible drug may help determine the severity of overdose and to determine the availability of antagonists. Intentional drug overdose is the most common method of self-harm. As psychiatric disorders are common in self-harm patients, the medication used to treat these disorders can become the means for the self-harm act. Therefore, this study focused on the administration of physician-prescribed medications before emergency transportation. Information regarding medicines prescribed before emergency transportation can be obtained at the time of transportation from the medical emergency personnel or from the patients’ families.

Although there are studies investigating the prescription status of psychotropic drugs before emergency transportation [12–19], few studies have focused on patients with disorders of consciousness. Moreover, as the potential risks associated with psychotropic drug overdose are known to be diverse depending on gender and age [12–19], it has been difficult to identify specific risks associated with overdose during emergency transport in patients with disorders of consciousness.

In this study, we investigated the association between drug overdose in transported patients with consciousness disorders and psychotropic medications (especially BZs and antidepressant drugs) administered before emergency transportation. Since the backgrounds differ depending on the age group, we investigated whether the types of related psychotropic drugs differed depending on the age group.

## Methods

### Patients and patient data

The study investigated a total of 222 patients diagnosed with disorder of consciousness, who had been transported to the Emergency and Critical Care Center of Teikyo University Hospital from October 19, 2016, to December 20, 2017 (Table 1). Of these, patients who were finally diagnosed with overdose by their doctors were defined as “overdose” patients. The patients were divided into an “overdose” group (*n* = 128) and “other consciousness disorders” group (*n* = 94). Overdose patients were further divided into three groups, according to age: ≤39 years (*n* = 72), 40–59 years (*n* = 34), and ≥ 60 years (*n* = 22). Similarly, patients with other consciousness disorders who were included in the control group were further divided into three groups, according to age: ≤39 years (*n* = 28), 40–59 years (*n* = 31), and ≥ 60 years (*n* = 35).

**Table 1:** Clinical characteristics of patients

A. Study population		
	Overdose	Other consciousness disorders
	*n* = 128	*n* = 94
Age (Average ± S.D.)	40.6 ± 18.4	51.9 ± 19.7
≤ 39 years	72	28
40–59 years	34	31
≥ 60 years	22	35
Female/Male (n)	95/33	34/60
≤ 39 years	56/16	5/23
40–59 years	24/10	11/20
≥ 60 years	15/7	18/17
Medical history^a^		
Medical department (n, [%])		
Psychiatry	68 [53.1]	14 [14.9]
Psychiatry and general medical department	29 [22.7]	10 [10.6]
(Psychiatry positive)	(97 [75.8])	(24 [25.5])
General medical department	20 [15.6]	51 [54.3]
None or unclear	11 [8.6]	19 [20.2]
Psychiatric disorders (n, [%])		
ICD-10		
F2 (Schizophrenia, schizotypal, and delusional disorders)	25 [19.5]	8 [8.5]
F3 (Mood [affective] disorders)	60 [46.9]	12 [12.8]
F4 (Neurotic, stress-related and somatoform disorders)	31 [24.2]	3 [3.2]
F5 (Behavioural syndromes associated with physiological disturbances and physical factors)	10 [7.8]	1 [1.1]
F6 (Disorders of adult personality and behaviour)	9 [7.0]	1 [1.1]
Others	20 [15.6]	4 [4.3]
Other disorders (n, [%])		
Cardiovascular disorder	17 [13.3]	17 [18.1]
Respiratory disorder	9 [7.0]	4 [4.3]
Other disorders	78 [60.9]	57 [60.6]
Unclear	9 [7.0]	3 [3.2]
None	3 [2.3]	15 [16.0]
Outcome (n, [%])		
Hospital discharge	107 [83.6]	57 [60.6]
Hospitalization	17 [13.3]	29 [30.9]
Death	0 [0]	7 [7.4]
Unclear	4 [3.1]	1 [1.1]
B. Prescribed drugs		
	Overdose	Other consciousness disorders
Psychotropic drugs (n)	87	32
Benzodiazepines (n, [%])	71 [55.5]	27 [27.6]
Antidepressants (n, [%])	40 [31.3]	10 [10.2]
Antipsychotics (n, [%])	54 [42.2]	17 [17.3]
Anticonvulsants (n, [%])	29 [22.7]	6 [6.1]
Lithium carbonate (n, [%])	13 [10.2]	1 [1.02]

ICD-10, International Statistical Classification of Diseases and Related Health Problems-10

^a^Medical history indicates total number

Patient data were retrieved from the electronic medical records. The research data comprised age, sex, medical history (medical department and disorder), outcome, and prescribed drugs. In all patients, an investigation of prescribed drugs was conducted for all drugs within the range described in the electronic medical record. Patients with pesticide poisoning, detergent poisoning, narcotic poisoning, and psychostimulant poisoning were excluded from the study. Psychiatric disorders were further investigated according to the International Statistical Classification of Diseases and Related Health Problems-10 (ICD-10) in a medical history survey. Based on the information available in medication notebooks and electronic medical records of interviews with patients and families, the drugs that had apparently been prescribed before arrival in the emergency room were tabulated as prescribed drugs. Anticonvulsants included sodium valproate, lamotrigine, and carbamazepine.

### Statistical analysis

Logistic regression analysis was performed using the JMP Pro. 13 (SAS Institute Inc., Cary, NC, USA). The significance level was set at *P* < 0.05.

## Results

### Characteristics of patients prescribed drugs for overdose

The average age of patients in the overdose group was 40.6 years (control: 51.9 years), and the number of women was more than twice that of men (Table 1). Nearly 80% of overdose patients had a history of psychiatric consultation, of which the most common psychiatric disorders were F3 (mood [affective] disorders), followed by F4 (neurotic, stress-related, and somatoform disorders), and F2 (schizophrenia, schizotypal, and delusional disorders) (Table 1). As for the outcome, more than 80% (107/128, control 57/94 60.6%) of the patients returned home early without hospitalization (Tables 1). The characteristics of prescribed psychotropic drugs in overdose patients were examined. As shown in Table 2, univariate analysis revealed that female sex (odds ratio [OR] 5.08, 95% confidence interval [CI] 2.9–9.1, *P* < 0.00001), psychotropic drugs (OR 4.11, 95% CI 2.34–7.24, *P* < 0.0001), BZs (OR 3.09, 95% CI 1.75–5.45, *P* < 0.0001), antidepressant drugs (OR 3.82, 95% CI 1.80–8.12, *P* = 0.0005), antipsychotic drugs (OR 3.31, 95% CI 1.76–6.22, *P* = 0.0002), lithium carbonate (OR 10.5, 95% CI 1.35–81.8, *P* = 0.0247), and anticonvulsants (OR 4.30, 95% CI 1.70–10.8, *P* = 0.002) were significantly correlated with overdose. According to multivariate logistic regression analysis (Table 2), female sex (OR 4.54, 95% CI 2.43–8.05, *P* < 0.0001) was associated with overall overdose.

**Table 2:** Univariate analysis and multivariate logistic regression for overdose (overall)

	Univariate analysis		Multivariate analysis	
	Crude odds ratio (95% CI)	*P* -value	Adjusted odds ratio (95% CI)	*P* -value
Female sex	5.08 (2.9–9.1)	< 0.0001^a^	4.54 (2.43–8.50)	< 0.0001^a^
Psychotropic drugs	4.11 (2.34–7.24)	< 0.0001^a^		
Benzodiazepines	3.09 (1.75–5.45)	< 0.0001^a^	0.97 (0.44–2.16)	0.94
Antidepressant drugs	3.82 (1.80–8.12)	0.0005^a^	2.09 (0.81–5.38)	0.13
Antipsychotic drugs	3.31 (1.76–6.22)	0.0002^a^	1.81 (0.81–4.07)	0.15
Lithium carbonate	10.5 (1.35–81.8)	0.0247^a^	5.06 (0.58–44.4)	0.14
Anticonvulsants	4.30 (1.70–10.8)	0.002^a^	2.39 (0.85–6.67)	0.10

Female sex: Overdose 95/128 (74.2%) and other consciousness disorders 34/94 (36.1%)

*CI* Confidence interval

^a^Significant difference

### Age-specific analysis of prescribed psychotropic drugs for overdose

To examine the characteristics of prescribed psychotropic drugs by age group, we classified the patients into three age groups: ≤39 years, 40–59 years, and ≥ 60 years (Table 3). As shown in Table 3, univariate analysis revealed that BZs (OR 4.64, 95% CI 1.61–13.4, *P* = 0.005) and antidepressant drugs (OR 11.4, 95% CI 2.35–55.8, *P* = 0.003) were significantly correlated with overdose. However, antipsychotic drugs did not significantly correlate with overdose in the 40–59 years age group. In contrast, the use of BZs, antidepressant drugs, and antipsychotic drugs was not significantly different in the ≤39 years and ≥ 60 age groups. Data of patients prescribed lithium carbonate were unsuitable for analysis owing to small sample size. Anticonvulsants were associated with overdose in the 40–59 years age group (Table 4), while data of patients in the ≤39 years and ≥ 60 years age groups were unsuitable for analysis owing to small sample size.

**Table 3:** Univariate logistic analysis for overdose of prescribed psychotropic drugs by age group

Age group	Univariate analysis Odds ratio (95% CI)	*P*-value
(a) Benzodiazepines		
≤ 39	2.36 (0.96–6.13)	0.07
40–59	4.64 (1.61–13.4)	0.005^a^
≥ 60	3.00 (0.98–9.14)	0.053
(b) Antidepressant	drugs	
≤ 39	1.61 (0.57–4.53)	0.364
40–59	11.4 (2.35–55.8)	0.003^a^
≥ 60	2.61 (0.40–17.0)	0.317
(c) Antipsychotic	drugs	
≤ 39	2.54 (0.96–6.72)	0.061
40–59	2.71 (0.92–7.97)	0.071
≥ 60	4.00 (0.88–18.1)	0.07

n: Overdose/Other consciousness disorders; ≦39 years (72/28), 40–59 years (34/31) and ≧60 years (22/35)

^a^Significant difference

**Table 4:** Univariate analysis and multivariate logistic regression for overdose in patients aged 40–59 years

	Univariate analysis		Multivariate analysis	
	Crude odds ratio (95% CI)	*P*-value	Adjusted odds ratio (95% CI)	*P*-value
Female sex	4.36 (1.54–12.4)	0.006^a^	4.44 (1.37–14.3)	0.013^a^
Psychotropic drugs	5.05 (1.75–14.6)	0.003^a^		
Benzodiazepines	4.64 (1.61–13.4)	0.005^a^	1.84 (0.48–7.08)	0.377
Antidepressant drugs	11.4 (2.35–55.8)	0.003^a^	7.95 (1.21–52.1)	0.031^a^
Antipsychotic drugs	2.71 (0.92–7.97)	0.071		
Anticonvulsants	4.46 (1.11–17.9)	0.035^a^		

Female sex: Overdose 24/36 (70.6%); other consciousness disorders 11/31 (35.5%)

n: Overdose/Other consciousness disorders; 40–59 years (34/31)

*CI* Confidence interval

^a^Significant difference

### Analysis of overdose in patients in the 40–59 years age group

According to univariate logistic regression analysis (Table 4), female sex (OR 4.36, 95% CI; 1.54–12.4, *P* = 0.006), psychotropic drugs (OR 5.05, 95% CI; 1.75–14.6, *P* = 0.003), BZs (OR 4.64, 95% CI; 1.61–13.4, *P* < 0.05), antidepressants (OR 11.4, 95% CI; 2.35–55.8, *P* = 0.003), and anticonvulsants (OR 4.46, 95% CI; 1.11–17.9, *P* = 0.035) were associated with overdose in patients in the 40–59 years age group.

Sex, BZs, and antidepressant drugs are known to be associated with overdose [1, 3]. In this analysis, female sex, BZs, and antidepressant drugs were selected for multivariate logistic analysis, finding that female sex (OR 4.44, 95% CI; 1.37–14.3, *P* = 0.013) and antidepressant drugs (OR 7.95, 95% CI; 1.21–52.1, *P* = 0.031) were associated with overdose in patients in the 40–59 years age group (Table 4).

## Discussion

In this study, we investigated the association between drug overdose in transported patients with consciousness disorders and psychotropic medications (especially BZs and antidepressant drugs, which have been reported to have different potential risk profiles depending on age) administered before emergency transportation. All prescription drugs in the subjects were analyzed, but only analysis of psychotropic drugs provided significant results in line with the objectives of this study; therefore, we focused on psychotropic medications. Multivariate analysis of overall age showed that female sex was associated with drug overdose in disorders of consciousness, and no other categories of psychotropic medications were found to be associated with drug overdose. Age-specific logistic analysis of BZs, antidepressants, and antiepileptic drugs showed that the use of BZs and antidepressants in patients aged 40–59 years was associated with disorders of consciousness due to drug overdose. Multivariate analysis of data of patients in the 40–59 years age group showed that female sex and use of antidepressants were associated with consciousness disorders due to overdose.

In this study, female sex was associated with drug overdose for all age groups. This finding is in line with those of the previous studies in which there were a greater number of females with overdose of psychotropic drugs and there was a higher suicide rate due to drug overdose in females, although males had a higher suicide rate and recurrence rate [17]. Furthermore, it has been reported that overdose of psychotropic drugs is associated with the prescription of psychotropic drugs before emergency transportation, with BZs accounting for 65% of prescriptions before transportation [24]. In addition, an increase in the odds of using antidepressants and antipsychotics in episodes of suicide was observed when the rate of prescription increased [17]. In this study, prescription of BZs, antipsychotics, and antidepressants was confirmed in patients with disorders of consciousness due to overdose. This finding was in agreement with the above results, although there were differences in the aggregation method.

Multivariate analysis of data of patients in the 40–59 years age group showed that female sex and use of antidepressants were associated with disorders of consciousness due to overdose, although there was no association with BZs. Previous studies have reported that antidepressants are significantly more commonly used for intentional drug overdose among women than among men [17–19]. In addition, regarding age dependency of the comparison of the rates of suicide attempts after and before the start of treatment with antidepressants, it was reported that the highest rates of suicide were observed among those > 40 years during the first month [25]. In this study, among overdose patients, there was a high proportion of F3 in patients with depression. Depression is also known to be the main clinical manifestation of menopause and often occurs in females aged 45–55 years. Previous studies have shown that in menopausal women, 50–60% have mild depression and 1–3% suffer from severe depression, among which about 15% have suicidal behavior [26]. Furthermore, with an increase in the use of antidepressants due to the onset of depression in menopause, it can be assumed that the risk of antidepressant use has increased in females in the 40–59 years age group.

BZs are widely prescribed in general clinical departments as well as for patients with psychiatric disorders, including children and the elderly [27]. A survey on BZ prescriptions indicated that 54% of the prescriptions, including BZs, were from departments other than psychiatry, and 33.8% were for patients over 65 years old [27]. Prescriptions for elderly patients from the psychiatry department were less frequent than those from other departments. In our study, BZs had lower odds than antidepressants according to multivariate analysis. The reason for this is that BZs are widely prescribed in general clinical departments other than psychiatry for sleep disorders. In this study, the proportions of overdose patients and other patients with impaired consciousness who visited the general clinical department were 58.3 and 64.3%, respectively, in the 40–59 years age group, and no significant difference was observed between the two groups. Therefore, the odds ratio of BZs may not have been as high as that of antidepressants in the 40–59 years age group.

Based on the results of this research, it is better to proactively identify drugs brought to the emergency outpatient department and to link them to research results, such as shortening the time required to diagnose the cause of consciousness disorders compared to before initiating response. Moreover, in terms of clinical practice, this study’s logistic analysis results may potentially indicate what should be noted when managing a patient’s medication. To prevent overdose, psychotropic drug optimization may be needed in patients in their 40–50s, although larger validation studies are needed.

The limitation of this study is that information on the time and period of prescription and prescription amount of each psychotropic drug was not available. Since the purpose of this study was to obtain an index from the information during emergency transportation of patients, we collected data regarding the presence or absence of prescription drugs during emergency transportation. In addition, owing to the small number of cases, it was not possible to classify and analyze antidepressants, such as tricyclic antidepressants, tetracyclic antidepressants, and selective serotonin reuptake inhibitors. In the future, we would like to increase the number of cases to determine the other highly relevant antidepressants.

## Conclusions

In this study, we were able to identify factors related to drug overdose in patients with disorders of consciousness depending on age. As a reference in distinguishing overdose in women in their 40s and 50s who present with impaired consciousness, attention may need to be paid to the type of psychotropic drug used, especially antidepressants.

## Data Availability

The datasets used and/or analyzed during the current study are available from the corresponding author on reasonable request.

## Abbreviations

BZ: Benzodiazepines
CI: Confidence interval
OR: Odds ratio
